# Editorial: The Non-coding Genome and Cardiovascular Disease

**DOI:** 10.3389/fcvm.2019.00098

**Published:** 2019-07-16

**Authors:** Paras Kumar Mishra, Georges Nemer

**Affiliations:** ^1^Department of Cellular and Integrative Physiology, University of Nebraska Medical Center, Omaha, NE, United States; ^2^Department of Anesthesiology, University of Nebraska Medical Center, Omaha, NE, United States; ^3^Department of Biochemistry and Molecular Genetics, American University of Beirut, Beirut, Lebanon

**Keywords:** miRNA, lncRNA, genetic mutation analysis, cardiomyopathies, diabetes, heart

Only ~1.5% of the genes in the human genome code for proteins, with the remaining 98.5% constitutes the non-coding genome (1). These non-coding RNAs range from 18–25 nucleotides (microRNA) to 200–300 nucleotides (long non-coding RNA or lncRNA) and play crucial roles in fine-tuning gene expression (2, 3). Empirical evidence demonstrates that these non-coding RNAs are differentially expressed in cardiovascular diseases (4). Studies on many preclinical models suggest that targeting these non-coding RNAs could be a promising therapeutic strategy for cardiovascular diseases (5, 6). As per the National Institutes of Health website: https://clinicaltrials.gov, there are 140 clinical trials carried out on “miRNA and cardiovascular diseases” and 10 clinical trials conducted on “lncRNA and cardiovascular diseases” to date, supporting the translational significance of non-coding RNAs in cardiovascular diseases. Being an endogenous molecule and having regulatory roles in several biological pathways, non-coding RNAs remain an attractive target in the field of cardiovascular disease as they hold promise as both therapeutic targets and biomarkers (7–9). The aim of the current special Research Topic was to understand the regulatory role and therapeutic potential of non-coding RNAs in cardiovascular diseases. Four articles (three reviews and one research) have been published in this Research Topic, which have elaborated the distinct roles of non-coding RNA in different biological signaling pathways related to cardiovascular diseases. The specific contributions of each articles are summarized below:

The first paper by Biswas et al. reviewed the key molecular mechanisms and their regulations by lncRNAs during fibrosis in the diabetic heart. Approximately 18,480 lncRNAs are present in the human heart and the majority of them are differentially expressed during cardiomyopathy (10). In a given cell, lncRNAs are localized to either the cytoplasm or nucleus in order to regulate gene expression by epigenetic modifications in the nucleus and mRNA translation by sponging miRNA in the cytoplasm (11). The authors summarized the role of different lncRNAs in cardiac complications, such as Mhrt and H19 in protecting the heart against hypertrophy, and Wisper in controlling cardiac fibrosis. In addition, Biswas et al. elaborated the key molecular players of extracellular matrix remodeling, endothelial-mesenchymal transition, and epigenetic regulatory mechanisms by DNA and histone methylation and histone acetylation that contribute to cardiac fibrosis in diabetes mellitus.

The second paper by Das et al. highlighted the important basic and clinical breakthroughs in the field of non-coding RNAs as a therapeutic target and biomarker for cardiovascular disease, and depicted the limitations and challenges of non-coding RNA-based therapeutics. They have extensively reviewed the role of different types of miRNAs in adverse cardiac remodeling and heart failure, myocardial ischemia/reperfusion injury, atherosclerosis, diabetes mellitus, and insulin signaling. The emerging roles of other non-coding RNAs such as siRNA, piwi-interacting RNA, circular RNA, and lncRNA are elaborated in this paper. The potential of non-coding RNA, especially miRNA, as a predictor, prognostic tool, and therapeutic target for cardiovascular diseases and the strategies to tackle critical barriers in translating miRNA therapeutics from bench to bedside are extensively discussed.

The third paper by Salman et al. reviewed the impact of genetic mutation on the non-coding part of the genome in different types of cardiomyopathies (CM), including dilated CM, hypertrophic CM, restrictive CM, arrhythmogenic right/left ventricular CM, and left ventricular non-compacted CM. They also summarized the role of intronic mutations in inherited cardiomyopathy. They have described in detail the impact of mutations in different genes on cardiomyopathy, including myosin binding protein C3, NF-kappa B inhibitor-like protein 1, C-C motif chemokine ligand 2/monocyte chemoattractant protein 1, spliceosome RNA helicase BAT1, 14-3-3 Epsilon, dystrophin, plakophilin-2, frataxin, dystrophia myotonica-1 protein kinase, and neurite outgrowth inhibitor proteins. They have elaborated the mutation in genes coding for in islet-1 transcription factor, promoter, enhancer, miRNAs, 3′ and 5′ untranslated regions, and lncRNA in different types of cardiomyopathy.

The fourth paper by Kambis et al. demonstrated the functional roles of miR-133a in diabetes mellitus-induced cardiac remodeling. The transcription of miR-133a is restricted to the cardiac and skeletal muscles (12). MiR-133a is the most abundant miRNA in the human heart (13). It protects the heart against adverse cardiac remodeling including structural remodeling such as hypertrophy (14) and fibrosis (15) and functional remodeling such as contractility of the heart (16). Decreased cardiac levels of miR-133a are reported in cardiomyopathy (14) including diabetic cardiomyopathy (17–19). However, whether the forced expression of miR-133a could prevent diabetes-induced adverse cardiac remodeling including the metabolic remodeling remained unclear. To determine this, Kambis et al. created a novel strain of diabetic mice where miR-133a was increased almost 2-fold only in the heart. By comparing hearts with increased miR-133a expression with diabetic heart with reduced miR-133a, they revealed that increased cardiac levels of miR-133a could prevent diabetes mellitus-induced cardiac hypertrophy, fibrosis, and lipid deposition (metabolic remodeling) without altering the blood glucose levels. They concluded that miR-133a overexpression could be a promising therapeutic candidate to prevent structural and metabolic remodeling in the diabetic heart. Although increasing the levels of miR-133a in the diabetic heart is cardioprotective, its anti-fibrotic effect may not be beneficial in atherosclerotic coronary vessels, where the fibrosis of the extracellular matrix stabilizes vascular lesions from rupture. In both stable and unstable angina, circulating miR-133a has increased levels (20). Reduced vascular flow in coronary artery causes myocardial infarction (MI). Plasma levels of miR-133a are elevated in patients with acute MI, suggesting that miR-133a could be a promising biomarker for acute MI (21). Overall, these findings suggest that tissue-specific upregulation of miR-133a is crucial for mediating its cardioprotective effects.

In conclusion, this Research Topic has provided fundamental new insights on the biogenesis and regulatory mechanisms of non-coding RNAs, as well as the impact of genetics and differential regulation of non-coding RNAs in cardiovascular diseases. It has also provided up-to-date knowledge on the potential of non-coding RNAs, especially miRNAs, as biomarkers and/or therapeutic candidates/targets for cardiovascular diseases. A majority of these biomarkers are still in early preclinical stages or clinical studies/trials. Finally, research findings that miR-133a prevents diabetes-induced metabolic and structural remodeling in the heart provides great therapeutic potential for cardiovascular diseases. The current challenges to non-coding RNA-based therapy for cardiovascular disease are pertaining to efficient and targeted delivery of the non-coding RNA into the specific region and/or cell type(s) of the heart. The heterogeneity of the disease poses another layer of challenge to the non-coding RNA-based therapy. For example, diabetes mellitus (DM)-induced cardiomyopathy could be due to insulin resistance (T2DM), insulin deficiency (T1DM), or both T1DM and T2DM (in advanced chronic DM) phenotype. The metabolic status of the heart differs in these three types of DM. Thus, targeting a particular non-coding RNA could have differential effects on these three types of DM heart. Thus, it will be important to determine the levels of specific non-coding RNA in the DM heart that correlate with the metabolic status of the DM heart. On the contrary, circulating levels of non-coding RNAs correlated with specific DM phenotypes and disease stages could serve as promising biomarkers for disease progression. Additionally, these biomarkers could be important for those diseases where limited methods are available to clinically diagnose them such as heart failure with preserved ejection fraction (HFpEF) (22). Non-coding RNA could be a promising biomarker to identify HFpEF and determine different stages of HFpEF. Thus, non-coding RNAs have tremendous potential as both biomarkers and therapeutic targets for cardiovascular diseases. The full potential of non-coding RNAs in treating cardiovascular diseases could be harnessed after developing strategies to restrict their off-target effects and improving their delivery to the target tissues and cell types.

## Author Contributions

PM drafted the manuscript. PM and GN reviewed and approved the manuscript.

### Conflict of Interest Statement

The authors declare that the research was conducted in the absence of any commercial or financial relationships that could be construed as a potential conflict of interest.

## References

[1] Lander ES. Initial impact of the sequencing of the human genome. Nature. (2011) 470:187–97. 10.1038/nature09792 21307931

[2] Fernandes JCR Acuna SM Aoki JI Floeter-Winter LM Muxel SM. Long non-coding RNAs in the regulation of gene expression: physiology and disease. Noncoding RNA. (2019) 5:17. 10.3390/ncrna5010017 30781588 PMC6468922

[3] Bartel DP Chen CZ. Micromanagers of gene expression: the potentially widespread influence of metazoan microRNAs. Nat Rev Genet. (2004) 5:396–400. 10.1038/nrg1328 15143321

[4] Duenas A Exposito A Aranega A Franco D. The role of non-coding RNA in congenital heart diseases. J Cardiovasc Dev Dis. (2019) 6:E15. 10.3390/jcdd6020015 30939839 PMC6616598

[5] Mishra PK Tyagi N Kumar M Tyagi SC. MicroRNAs as a therapeutic target for cardiovascular diseases. J Cell Mol Med. (2009) 13:778–89. 10.1111/j.1582-4934.2009.00744.x 19320780 PMC3822884

[6] Gurha P. Noncoding RNAs in cardiovascular diseases. Curr Opin Cardiol. (2019) 34:241–5. 10.1097/HCO.0000000000000615 30789852 PMC6459692

[7] Shah P Bristow MR Port JD. MicroRNAs in heart failure, cardiac transplantation, and myocardial recovery: biomarkers with therapeutic potential. Curr Heart Fail Rep. (2017) 14:454–64. 10.1007/s11897-017-0362-8 28940102

[8] Aufiero S Reckman YJ Pinto YM Creemers EE Circular RNAs open a new chapter in cardiovascular biology. Nat Rev Cardiol. (2019) 5:2 10.1038/s41569-019-0185-2 30952956

[9] Kok FO Baker AH. The function of long non-coding RNAs in vascular biology and disease. Vascul Pharmacol. (2019) 114:23–30. 10.1016/j.vph.2018.06.004 30910128

[10] Yang KC Yamada KA Patel AY Topkara VK George I Cheema FH. Deep RNA sequencing reveals dynamic regulation of myocardial noncoding RNAs in failing human heart and remodeling with mechanical circulatory support. Circulation. (2014) 129:1009–21. 10.1161/CIRCULATIONAHA.113.003863 24429688 PMC3967509

[11] Quinn JJ Chang HY. Unique features of long non-coding RNA biogenesis and function. Nat Rev Genet. (2016) 17:47–62. 10.1038/nrg.2015.10 26666209

[12] Liu N Bezprozvannaya S Williams AH Qi X Richardson JA Bassel-Duby R. microRNA-133a regulates cardiomyocyte proliferation and suppresses smooth muscle gene expression in the heart. Genes Dev. (2008) 22:3242–54. 10.1101/gad.1738708 19015276 PMC2600761

[13] Leptidis S El Azzouzi H Lok SI de Weger R Olieslagers S Kisters N. A deep sequencing approach to uncover the miRNOME in the human heart. PLoS ONE. (2013) 8:e57800. 10.1371/journal.pone.0057800 23460909 PMC3583901

[14] Care A Catalucci D Felicetti F Bonci D Addario A Gallo P. MicroRNA-133 controls cardiac hypertrophy. Nat Med. (2007) 13:613–8. 10.1038/nm1582 17468766

[15] Matkovich SJ Wang W Tu Y Eschenbacher WH Dorn LE Condorelli G. MicroRNA-133a protects against myocardial fibrosis and modulates electrical repolarization without affecting hypertrophy in pressure-overloaded adult hearts. Circ Res. (2010) 106:166–75. 10.1161/CIRCRESAHA.109.202176 19893015 PMC2804031

[16] Nandi SS Zheng H Sharma NM Shahshahan HR Patel KP Mishra PK. Lack of miR-133a decreases contractility of diabetic hearts: a role for novel cross talk between tyrosine aminotransferase and tyrosine hydroxylase. Diabetes. (2016) 65:3075–90. 10.2337/db16-0023 27411382 PMC5033264

[17] Chen S Puthanveetil P Feng B Matkovich SJ Dorn GW II Chakrabarti S. Cardiac miR-133a overexpression prevents early cardiac fibrosis in diabetes. J Cell Mol Med. (2014) 18:415–21. 10.1111/jcmm.12218 24428157 PMC3955148

[18] Kesherwani V Shahshahan HR Mishra PK. Cardiac transcriptome profiling of diabetic Akita mice using microarray and next generation sequencing. PLoS ONE. (2017) 12:e0182828. 10.1371/journal.pone.0182828 28837672 PMC5570368

[19] Nandi SS Duryee MJ Shahshahan HR Thiele GM Anderson DR Mishra PK. Induction of autophagy markers is associated with attenuation of miR-133a in diabetic heart failure patients undergoing mechanical unloading. Am J Transl Res. (2015) 7:683–96. 26064437 PMC4455344

[20] Feinberg MW Moore KJ. MicroRNA regulation of atherosclerosis. Circ Res. (2016) 118:703–20. 10.1161/CIRCRESAHA.115.306300 26892968 PMC4762069

[21] Peng L Chun-guang Q Bei-fang L Xue-zhi D Zi-hao W Yun-fu L. Clinical impact of circulating miR-133, miR-1291 and miR-663b in plasma of patients with acute myocardial infarction. Diagn Pathol. (2014) 9:89. 10.1186/1746-1596-9-89 24885383 PMC4082297

[22] Oktay AA Shah SJ. Diagnosis and management of heart failure with preserved ejection fraction: 10 key lessons. Curr Cardiol Rev. (2015) 11:42–52. 10.2174/1573403X09666131117131217 24251461 PMC4347209

